# Micro-Nano Surface Characterization and Bioactivity of a Calcium Phosphate-Incorporated Titanium Implant Surface

**DOI:** 10.3390/jfb12010003

**Published:** 2021-01-07

**Authors:** Fausto Zamparini, Carlo Prati, Luigi Generali, Andrea Spinelli, Paola Taddei, Maria Giovanna Gandolfi

**Affiliations:** 1 Laboratory of Biomaterials and Oral Pathology, School of Dentistry, Department of Biomedical and Neuromotor Sciences, University of Bologna, 40126 Bologna, Italy; fausto.zamparini2@unibo.it (F.Z.); andrea.spinelli4@unibo.it (A.S.); 2 Endodontic Clinical Section, School of Dentistry, Department of Biomedical and Neuromotor Sciences, University of Bologna, 40126 Bologna, Italy; carlo.prati@unibo.it; 3 Department of Surgery, Medicine, Dentistry and Morphological Sciences with Transplant Surgery, Oncology and Regenerative Medicine Relevance, University of Modena and Reggio Emilia, 41121 Modena, Italy; luigi.generali@unimore.it; 4 Biochemistry Unit, Department of Biomedical and Neuromotor Sciences, University of Bologna, 40126 Bologna, Italy; paola.taddei@unibo.it

**Keywords:** dental implant surfaces, ESEM-EDX, SEM-FEG, bioactivity, micro-Raman spectroscopy

## Abstract

The surface topography of dental implants and micro-nano surface characterization have gained particular interest for the improvement of the osseointegration phases. The aim of this study was to evaluate the surface micro-nanomorphology and bioactivity (apatite forming ability) of Ossean^®^ surface, a resorbable blast medium (RBM) blasted surface further processed through the incorporation of a low amount of calcium phosphate. The implants were analyzed using environmental scanning electronic microscopy (ESEM), connected to Energy dispersive X-ray spectroscopy (EDX), field emission gun SEM-EDX (SEM-FEG) micro-Raman spectroscopy and X-ray photoelectron spectroscopy (XPS) before and after immersion in weekly refreshed Hank’s balanced salt solution (HBSS) for 28 days. The analysis of the samples before immersion showed a moderately rough surface, with micropits and microgrooves distributed on all of the surface; EDX microanalysis revealed the constitutional elements of the implant surface, namely titanium (Ti), aluminum (Al) and vanadium (V). Limited traces of calcium (Ca) and phosphorous (P) were detected, attributable to the incorporated calcium phosphate. No traces of calcium phosphate phases were detected by micro-Raman spectroscopy. ESEM analysis of the implant aged in HBSS for 28 days revealed a significantly different surface, compared to the implant before immersion. At original magnifications <2000×, a homogeneous mineral layer was present on all the surface, covering all the pits and microgrooves. At original magnifications ≥10,000×, the mineral layer revealed the presence of small microspherulites. The structure of these spherulites (approx. 2 µm diameter) was observed in nanoimmersion mode revealing a regular shape with a hairy-like contour. Micro-Raman analysis showed the presence of B-type carbonated apatite on the implant surface, which was further confirmed by XPS analysis. This implant showed a micro-nano-textured surface supporting the formation of a biocompatible apatite when immersed in HBSS. These properties may likely favor bone anchorage and healing by stimulation of mineralizing cells.

## 1. Introduction

A direct bone apposition onto the surface of titanium is critical for the successful integration of the implant, and the long-term success of the rehabilitation. Indeed, several biological processes start immediately after implant insertion [1], which follows the traditional intrabony wound healing phases: hemostasis (minutes to first hours), inflammatory phase (first hours to days), proliferative days (days to three weeks) and remodeling phase (from three weeks to years) [2].

An essential role in osseointegration processes during this period is played by the surface topography of the dental implants [3]. The micro and nano structures of the implants in contact with the bone tissues significantly enhance bone apposition, through a higher bone-to-implant contact [4], osteoblast cells attachment proliferation, and differentiation [5].

Topographically modified titanium surfaces such as sandblasted, large-grit, acid-etched implants are already implemented clinically, showing successful performances [6]. Calcium phosphates (CaPs) such as hydroxyapatite, beta-tricalcium phosphate and mixtures have been considered attractive blasting materials, being resorbable, biocompatible, osteoconductive and bioactive [7,8].

When considering implants surface modifications, these could be achieved though addition or subtraction methods [9]. Additive methods imply the addition of another material/compound, which is added onto the surface (implant coating) or integrated within the titanium oxide layer (incorporation process) [10].

However, the addition of a CaP coating revealed several drawbacks in the past years, including its dissolution after surgical procedures, failed interfacial adhesion between implant and CaP layer and subsequently higher early implant failure [11,12].

Ossean^®^ is a moderately rough Ti-Al-V surface obtained through the resorbable blasted medium (RBM) process and followed by the incorporation of a low amount of CaP.

The surface roughness was conceived to obtain micro and nano irregularities, which should enhance implant biocompatibility compared to traditional surfaces, increasing the available contact surface and potentially improving the mineralizing cells attachment and expansion [13].

The incorporation of a low amount of CaP may improve the surface biointeractivity during the initial osseointegration processes through the apatite nucleation on its surface without the abovementioned detrimental effects; however, no information on the apatite nucleation ability of the implant has been reported.

The evaluation of the apatite-forming ability of implant materials in simulated body fluids (SBF) is useful for the assessment of their biointeractivity (ability to exchange information with a biological system) and bioactivity (ability to cause a positive reaction in the host tissues), as well as to predict their in vivo bone-bonding ability [14,15].

The aim of this study was to analyze the surface micro and nano/morphology of Ossean^®^ surface and its modifications after immersion in SBF. The null-hypothesis of the present study was that there is no difference in the superficial elemental composition of a Ti-Al-V implant treated with a resorbable blasted medium process before and after immersion in Hank’s balanced salt solution.

## 2. Materials and Methods

### 2.1. Implants

Two implants were received in the commercial packaged and sterilized form (Intralock, Boca Raton, FL, USA) (lot: ak980, exp. Date 2018-10; lot: ak991, exp. Date 2018-10).

### 2.2. Surface Micro and Nano Characterization and CaPs Nucleation in Simulated Body Fluids (SBF)

The International Standard ISO 23317 method (BS ISO 23317, 2007) was used to evaluate the formation of a layer rich in Ca and P on the surface of the implants soaked in Hank’s balanced salt solution (HBSS), which was used as SBF according to several studies [16,17,18,19,20]. The HBSS (Cambrex Bio Science Verviers, Belgium) composition was: Ca^2+^ 1.27 mM, Cl^−^ 144.7 mM, K^+^ 5.8 mM, Na^+^ 141.6 mM, Mg^2+^ 0.81 mM, HCO_3_^−^ 4.17 mM, SO_4_^2−^ 0.81 mM, H_2_PO_4_^−^ 0.44 mM and HPO_4_^2−^ 0.336 mM.

One sample was analyzed before immersion. The other sample was placed vertically (BS ISO 23317, 2007) in 20 mL of HBSS at 37 °C. The medium was weekly refreshed for 28 days. At this endpoint, the surface was non-invasively examined by ESEM-EDX and micro-Raman spectroscopy to assess the formation of CaP.

#### 2.2.1. ESEM-EDX Microanalysis

Microanalyses were performed using an environmental scanning electron microscope (ESEM-Quanta 200, Fei Company-Oxford Instruments, Eindhoven, NL, USA) connected to a secondary electron detector for energy dispersive X-ray spectroscopy (EDS; INCA, Oxford Instruments, Oxford, UK) using computer-controlled software [16,17,18,19,20,21]. The whole samples were examined without sputtering at low vacuum (100 Pascal), accelerating voltage of 20 kV, working distance 8.5 mm, 0.5 wt% detection level, 133 eV resolution, amplification time 100 μs, measuring time: 60 s for spectra. Standard acquisition resolution was 1536 × 1024.

Then, EDX microanalyses were carried out at 2000× original magnification at random areas of ~50 × 50 μm to evaluate the relative element content. The elemental microanalysis (weight % and atomic %) with ZAF correction method, a procedure in which corrections for atomic number effect (Z), absorption (A), and fluorescence (F) are calculated separately, was performed in full frame and spot mode to analyze entire areas or specific points, respectively [19,20,21].

#### 2.2.2. FEG-SEM-EDX

Surface characterization of the implant surface on the implant before immersion and after 28 days immersion in HBSS solution was performed by using a field emission gun scanning electron microscope (FEG-SEM: Nova NanoSEM 450; FEI Company-Oxford Instruments, Eindhoven, NL, USA). Samples were observed without sputtering with the following parameters: accelerating voltage of 12 kV, working distance 6.0–6.5 mm, 133 eV resolution, amplification time 100 μs, measuring time: 60 s for spectra. Standard acquisition resolution was 1536 × 1024.

EDX (Quantax-200 system with XFlash 6/10 Si-drift detector: Bruker Corp., Billerica, MA, USA) spot microanalyses were performed at 500–2000× and 10,000–200,000× original magnifications. Areas of ~30 × 30 μm were selected for images at 2000× original magnifications, while areas of ~2 × 2 μm at original magnifications ≥10,000× were investigated.

#### 2.2.3. Raman Spectroscopy and XPS Analysis

Micro-Raman spectra were obtained by using an NRS-2000C (Jasco International Co. Ltd., Tokyo, Japan) instrument with a microscope of 100× original magnification. All the spectra were recorded in back-scattering conditions with 5 cm^−1^ spectral resolution by using the 532 nm green diode-pumped solid-state laser driver (RgBLase LLC, Fremont, CA, USA). A 160 K cooled digital charge coupled device (Spec-10: 100B, Roper Scientific Inc., Sarasota, FL, USA) was used as a detector. Laser power on the sample was about 10 mW for the implant before immersion and about 20 mW for the HBSS-aged sample. To obtain a good representation of the analyzed implants, 8–10 micro-Raman spectra were collected in different points of each sample.

XPS was performed on the implant before and after immersion for 28 days in HBSS to investigate the surface modification and apatite nucleation ability. A hemispherical energy analyzer (9 channeltron Phoibos HSA3500 150, SPECS GmbH, Berlin, Germany) was used. X-ray source was MgKα emission line (1253.6 eV) with an incidence of 45°. Samples were analyzed without any treatment at 7 × 10^−10^ mbar pressure. Data were acquired with LabSpecs and analyzed with Igor Pro 6.37 software.

## 3. Results

### 3.1. Implant before Immersion

#### 3.1.1. ESEM-EDX Analysis

**Figure 1:**
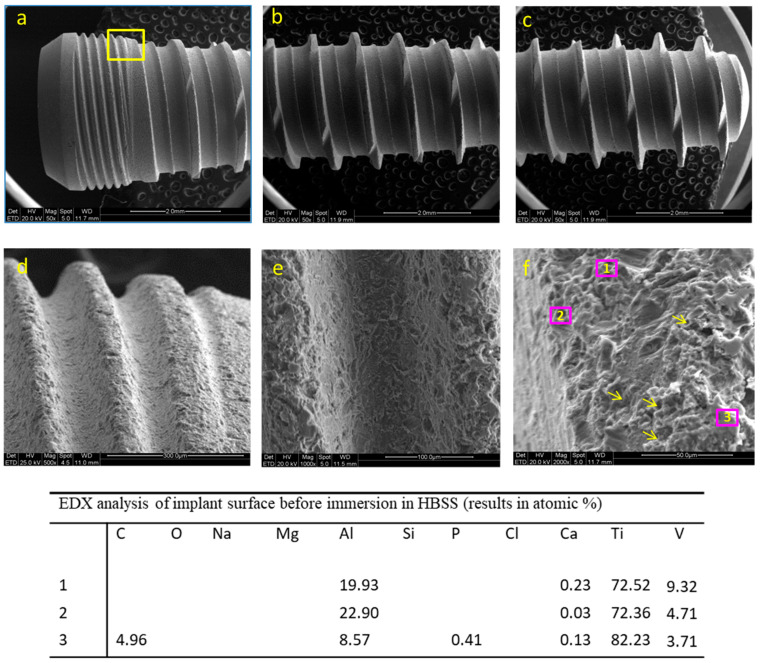
ESEM images of the coronal (**a**), medium (**b**) and apical (**c**) portion of the implant before immersion. EDX analyses (**f**) performed in five regions of interest; the pink square mark represents the area observed at progressively higher magnification. Thread at the collar portion at 500× (**d**), 1000× (**e**) and 2000× (**f**) original magnifications. EDX microanalysis is of on one thread located on the medium portion of the implant before immersion. Pink numbered squares represent the random areas analyzed through EDX. In addition to the constitutional elements of the Ti-Al-V dental implant surface, very low traces of Ca and P were detected. Arrows indicate the presence of pits and craters on the implant surface.

ESEM images of the coronal, medium and apical portion of the implant before immersion are reported in Figure 1a–c. A tapered configuration with regular threads is revealed. Tapping segments, designed to reduce the stress during the implant insertion procedures, are present along all the threads. The images of one thread at the collar portion at 500×, 1000× and 2000× original magnifications are reported in Figure 1d–f. The analysis revealed a moderately rough surface, with irregular pits and craters. The structures identified were comprised between 2 and 10 microns and were uniformly distributed on all of the implant surface.

EDX microanalysis revealed the elements constituting the implant surface, namely titanium (Ti), aluminum (Al) and vanadium (V). Very low traces of calcium (Ca) (detected on all the spectra) and phosphorous (P) (detected only in one spectrum) were detected (Figure 1).

#### 3.1.2. FEG-SEM-EDX Analysis

**Figure 2:**
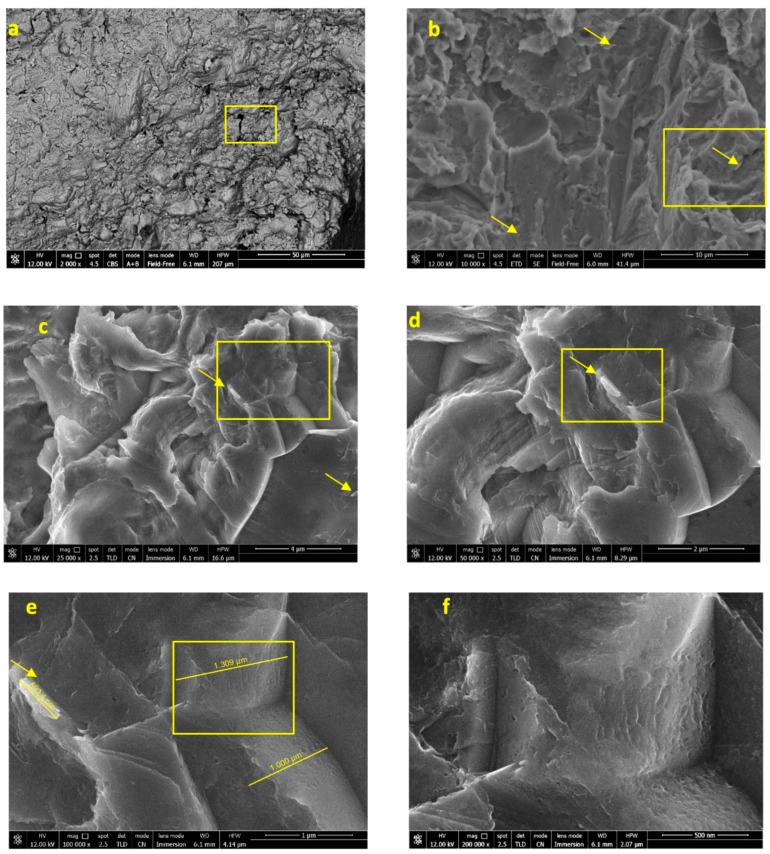
FEG-SEM images of the implant before immersion taken of one random thread located on the implant collar. Yellow square marks represent the areas observed at progressively higher magnifications (**a**) In backscattering mode, the pits and micro-irregularities of the moderately rough surface are well evident (2000× original magnification). (**b**) At 10,000× original magnification, some crystal rods may be observed (arrows). One area was observed in nanoimmersion mode at 25,000× (**c**), 50,000× (**d**) and 100,000× (**e**) original magnifications. Frame at original 200,000×: (**f**) the pits and microgrooves were not smooth.

FEG-SEM images of the as-received implant are reported in Figure 2a–f. The surface appeared similar on all of the implant body, with no differences between the coronal, medium and apical portions. One thread located in the same region as the previous ESEM analysis was selected and observed at progressively higher magnifications. At 10,000× original magnifications, some crystal rods may be observed. The presence of these crystals may likely be attributed to the CaP incorporation. One area with a well-identifiable rod (measuring approx. 600 nm in length and 200 nm in width) was observed by using nanoimmersion mode at 25,000×, 50,000×, 100,000× and 200,000× original magnifications. 

The pits, observed at high magnifications, were not smooth, but revealed homogeneous nanorough structures, such as nanopits and nanogrooves uniformly distributed along all of the surface; the range of these structures was between 30 and 100 nm.

**Figure 3:**
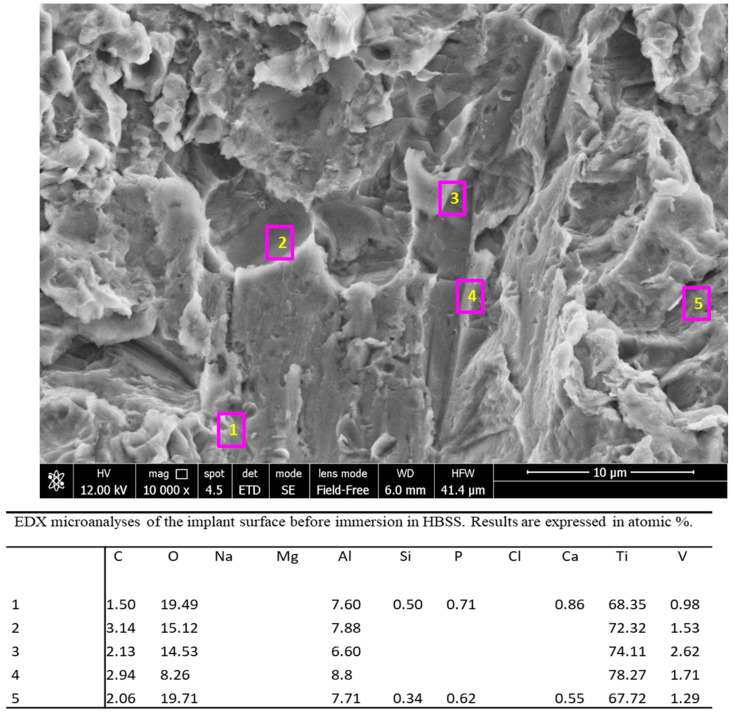
EDX microanalyses taken at 10,000× original magnification in five regions of interest (shown by numbered pink squares) of one randomly chosen area of the implant before immersion. The analyses revealed the constitutional elements of the tested implant (Ti, Al, V), O from the TiO_2_ layer. Presence of C and Si was also detected in low concentration.

EDX spectra (Figure 3) taken in five regions of interest at 10,000× original magnification revealed the presence of Ca and P only on the areas with the crystal rods previously identified. Interestingly, these sites also revealed the presence of Si. Spectra taken on the implant surface revealed the presence of Ti, Al, V (attributable to the dental implant alloy), O (attributable to the titanium oxide layer on the surface), and C.

#### 3.1.3. Raman Spectroscopy and XPS Analysis

**Figure 4:**
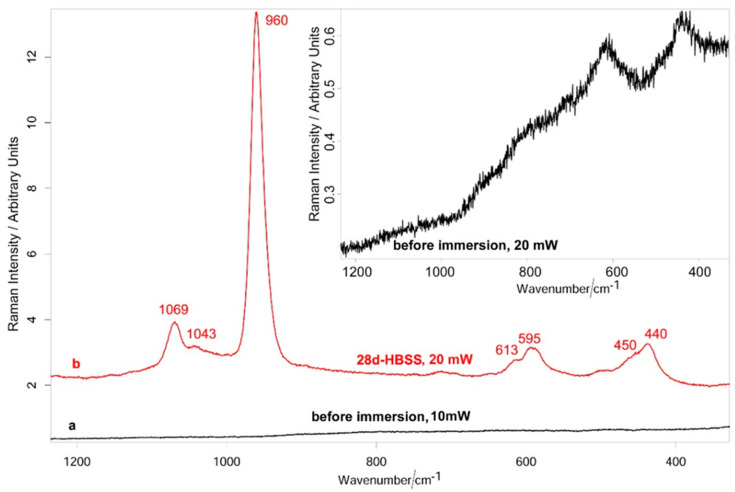
Average micro-Raman spectra: (a) recorded on the implant before immersion (100× magnification and laser power at the sample of 10 mW), and (b) after ageing in HBSS for 28 days (100× magnification and laser power at the sample of 20 mW). The spectrum in the inset was recorded on the implant before immersion at 100× magnification and laser power at the sample of 20 mW (i.e., under the same conditions as the aged implant). All the spectra are reported with their original intensity (no scaling-up was performed).

Figure 4 (spectrum a) shows the average micro-Raman spectrum recorded on the implant before immersion at 100× magnification and laser power at the sample of 10 mW. No bands were observed under these spectral conditions, according to the prevalently metallic composition of the sample (metals have no active vibrational Raman bands); at higher laser powers (i.e., 20 mW), the bands typical of Ti oxide as rutile polymorphic form were observed, due to sample degradation under laser exposure (Figure 4, inset). No CaP component was detected, due to its low concentration.

**Figure 5:**
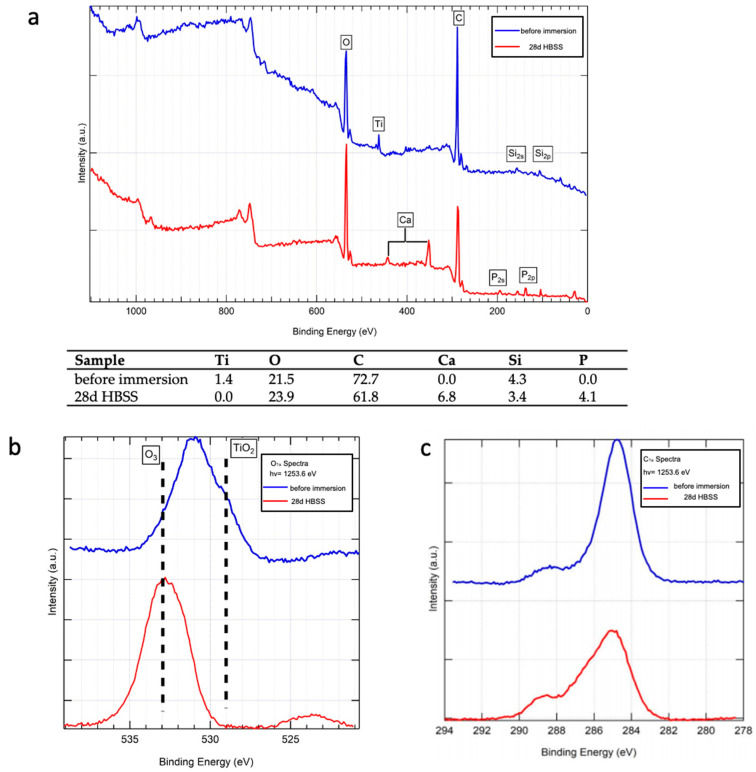
XPS analysis of the implant surface before immersion (blue trace) and after 28 days immersion in HBSS (red trace). The table reports the quantitative atomic percentage of elements on the implant surface of the sample before immersion and after immersion in HBSS. (**a**) Spectra acquisition using 2.0 eV step; (**b**) focused spectra using the same pass energy but 0.1 eV step at the oxygen site; (**c**) focused spectra using same pass energy but 0.1 eV step at the carbon site.

These data were further confirmed by the XPS analysis, which revealed the presence of Ti oxide peaks with limited and no traces of Ca and P (Figure 5).

### 3.2. Implants Soaked in HBSS (Time 28 Days)

#### 3.2.1. ESEM-EDX Analysis

**Figure 6:**
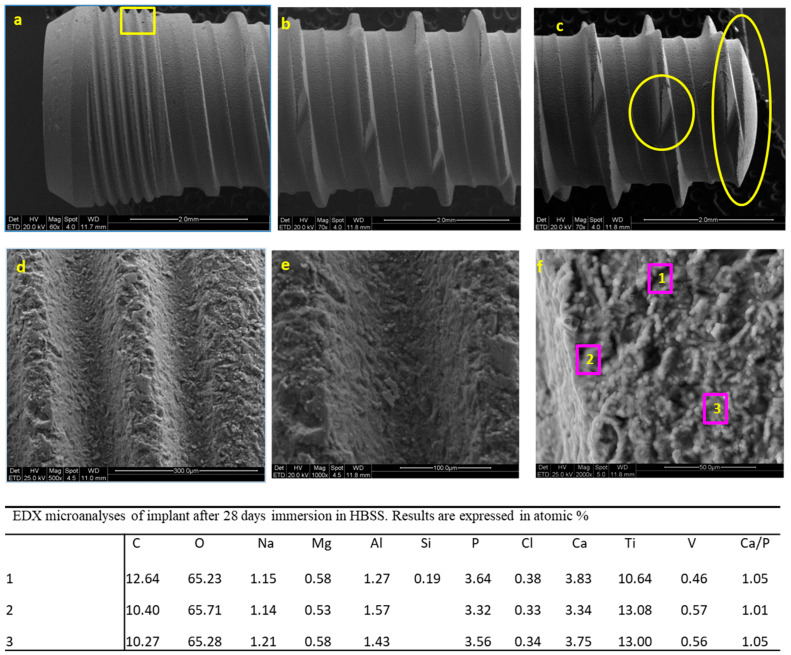
ESEM images taken at the coronal (**a**), medium (**b**) and apical (**c**) portion after 28 days immersion in HBSS. A homogeneous mineral layer was present on all of the implant surface. One random area taken at the implant collar was progressively observed at 500× (**d**), 1000× (**e**) and 2000× (**f**) original magnifications. EDX microanalysis was performed in three randomly chosen regions of one implant thread located in the coronal portion (2000× original magnification).

ESEM microanalysis of the implant soaked for 28 days in HBSS was carried out on all of the implant surface. Figure 6a–c reports the images taken at the coronal, medium and apical portion after 28 days immersion in HBSS. The surface appeared significantly different compared to the implant before immersion. A homogeneous mineral layer was present on all of the surface, covering all the pits and the microgrooves. Images of one coronal thread, which was in the same location as the implant before immersion, were analyzed at progressively higher magnifications. The micromorphology of the mineral layer revealed numerous regular spherical structures (Figure 6d–f).

As the mineral deposit was present on all of the surface, EDX microanalysis was performed on three randomly chosen regions of one thread located in the medium portion at 2000× original magnification (Figure 6). The analysis revealed similar results: the presence of Na, Mg, Cl, C, O (attributable to the HBSS medium), a general decrease of Ti, Al, V, (attributable to the presence of the mineral layer that covered the implant surface), an increase of Ca and P (attributable to the CaP layer). Ca/P atomic ratios calculated on sites 1–3 ranged from 1.01 to 1.05 (i.e., were 1.05, 1.01 and 1.05, respectively).

#### 3.2.2. FEG-SEM-EDX Analysis

**Figure 7:**
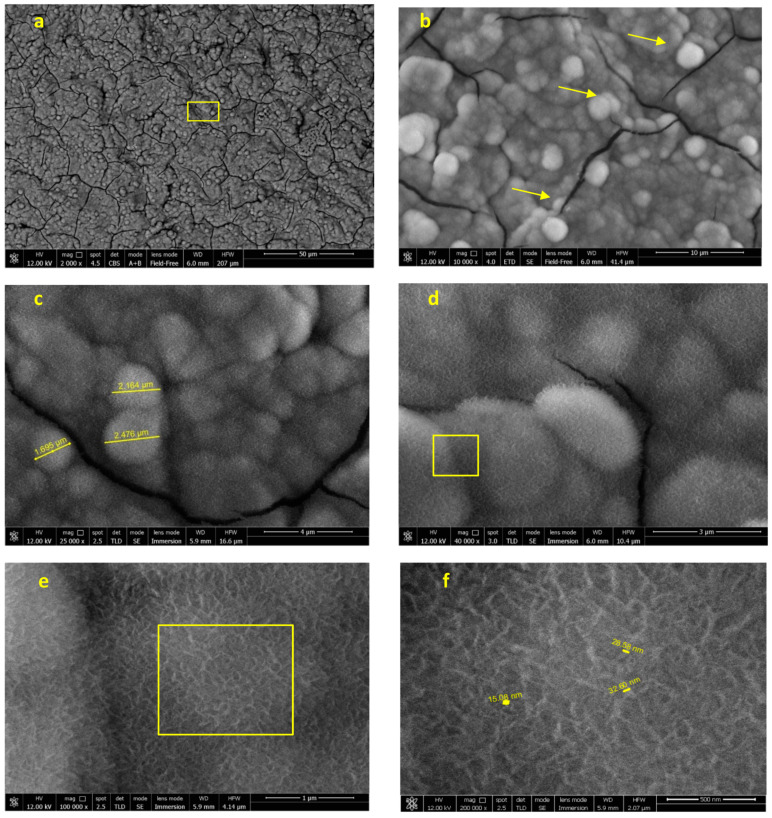
FEG-SEM images of the implant after 28 days of immersion in HBSS. One area in the coronal portion as investigated. Yellow square marks represent the areas observed at progressively higher magnifications: (**a**) Images at 2000× original magnification revealed that the mineral layer is uniform in all the investigated area. The pits and microgrooves are now covered and undetectable; the surface appears markedly different when compared to the implant before immersion. (**b**) The mineral layer revealed the presence of small circular structures (microspherulites, evidenced by arrows). (**c**,**d**) The morphology of these spherulites was observed at 25,000× and 40,000× original magnification. Needle-like crystals (width approx. 30 nm) can be identified at 100,000× (**e**) and 200,000× (**f**) original magnification.

FEG-SEM analysis carried out at the top of the previously observed coronal thread after 28 days immersion in HBSS was focused on one random area in the coronal portion (Figure 7). At low magnification (Figure 7a,b), a homogeneous mineral layer is displayed on all of the surface, markedly different from the implant before immersion. At high magnifications, the microspherulites were more deeply investigated. Some microcracks, attributable to the SEM high vacuum, were also identified. The structure of these spherulites (approx. 2 µm diameter) was observed in nanoimmersion mode (Figure 7c–f) revealing a regular shape with a hairy-like contour. These needle-like crystals (width approx. 30 nm) were better observed at 100,000× and 200,000× original magnifications.

**Figure 8:**
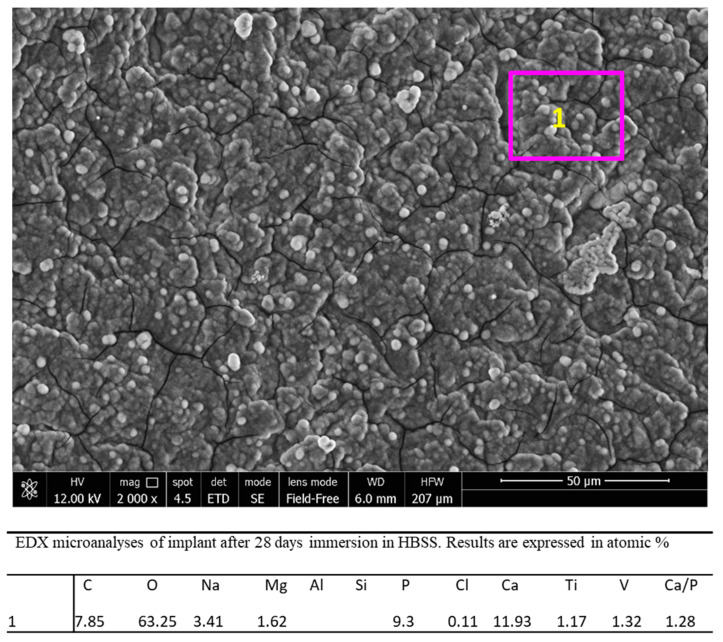
EDX microanalysis performed at 2000× original magnification on a randomly chosen area of the implant immersed in HBSS for 28 days.

EDX microanalysis (Figure 8) at 2000× original magnification revealed that the elements attributable to the implant surface, such as Ti and V, markedly decreased, while Al became undetectable. A notable increase of Ca and P and the appearance of Na, Cl, K, C and O (attributable to HBSS) were also detected, confirming the presence of a uniform mineral thin layer on the area. Ca/P atomic ratio was 1.28.

**Figure 9:**
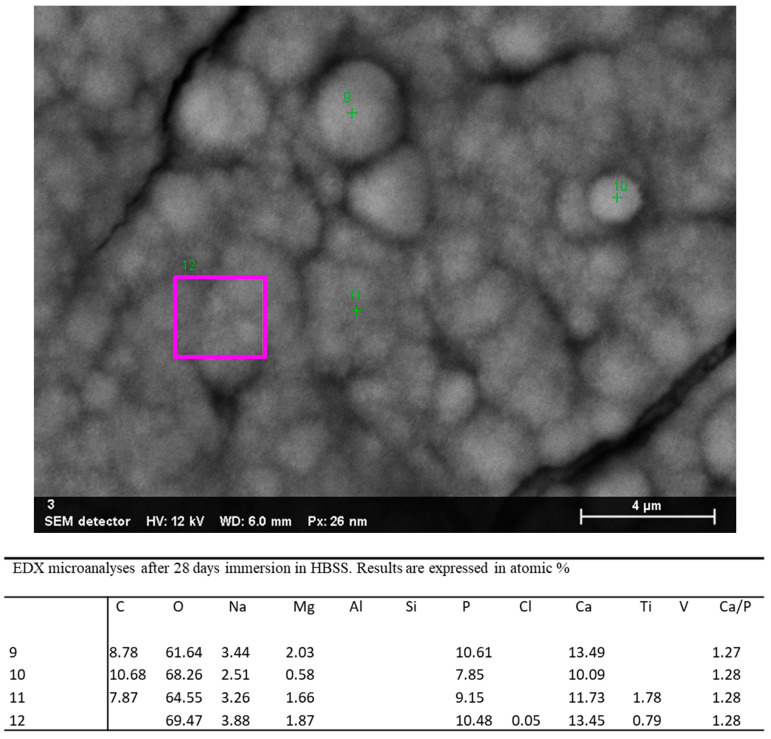
EDX microanalysis (at 25,000× original magnification) of a randomly chosen layer of microspherulites on the implant surface after 28 days of immersion in HBSS.

EDX microanalysis was performed at 25,000× original magnification on a randomly chosen layer of microspherulites (Figure 9). The analysis revealed the marked decrease of the elements attributable to the Ti-Al-V surface (Al and V were not detected, while Ti was observed only in 2 of 4 sites), and the presence of Na, Cl, Mg, C and O (attributable to HBSS solution). Ca/P atomic ratios were calculated on sites 9–12 and ranged from 1.27 to 1.28 (1.27,1.28, 1.28 and 1.28).

#### 3.2.3. Raman Spectroscopy and XPS Analysis

Figure 4 (spectrum b) shows the average micro-Raman spectrum recorded on the implant aged in HBSS. All the ten recorded spectra were analogous to those reported in the figure, confirming the homogeneous nature of the deposit. Bands at 1069, 1043, 960, 613–595, 450–440 cm^−1^, assignable to B-type carbonated apatite [18], were observed. To obtain a higher signal-to-noise ratio, the spectra were recorded under a laser power at the sample of 20 mW (i.e., higher than for the sample before immersion, analyzed at 10 mW); no bands of the rutile phase were detected under these conditions. XPS (Figure 5) confirmed the micro-Raman findings. The spectrum of the 28 d aged sample (Figure 5a) showed the presence of Ca and P peaks on the implant surface; at the same time, the Ti peak disappeared (see also the Table reported in Figure 5) together with the TiO_2_ O_1s_ peak (Figure 5b). The positions of the O_1s_ (Figure 5b) and C_1s_ (Figure 5c) peaks are consistent with those reported in the literature for carbonated apatites [22,23].

## 4. Discussion

In the present study, an implant surface characterization was performed by using repeatable, non-destructive methodologies (except desiccation artifacts due to vacuum), as samples did not require any manipulation, preparation or paraffin embedment. ESEM allowed visualization of the changes in the surface micro-topography, while SEM-FEG allowed us to assess the changes and nanotopography of the surface. The latter technique allows achievement of higher resolutions with lowered accelerating voltages.

HBSS solution was chosen, being a commercially available standardized soaking medium; it contains a lower calcium amount than human plasma, but the same phosphate concentration (mimicking human blood plasma). A 28-day immersion period was selected —in agreement with numerous studies investigating the biomaterials apatite nucleation ability—as this time represents the endpoint of a series of chemical reactions that lead to the formation of apatite or apatite precursors [14,15,16,17,19,20,24,25]. Indeed, a series of in vitro studies followed this protocol to analyze several experimental biomaterials, dental implant surfaces and bone tissue engineering scaffolds [14,15,16,17,19,20,24,25,26].

Surface reactivity and apatite nucleating ability of the present implant surface have been never investigated, and may provide useful information regarding the most suitable clinical use of this implant; a particularly bioactive surface stimulates cell attachment, differentiation and bone matrix synthesis [27], leading to an increased bone-implant contact in a shorter time, accelerating the process of bone-implant contact formation, and providing the implant with increasing secondary stability at the earliest stages of healing [18]. Thus, a highly reactive surface may be useful when complex cases are approached, namely when the implant is placed in endodontic post-extractive sites [28], where the implant is anchored only at the apical sites (such is the case of immediate post-extractive implants) [29,30], or when low density bone is present [11].

To obtain a reactive and osteoconductive surface, addition of a CaP coating has been studied and marketed in the past. However, these efforts were undermined by several complications, including peri-implantitis susceptibility, hydroxyapatite dissolution after surgery, and failed interfacial adhesion between implant and hydroxyapatite [11,12]. For these reasons, the current trend is to prefer to actively blast implant surface with bioactive compounds, modifying the surface micro and nano-topography, rather than provide a uniform bioactive layer a priori [6,11].

In the present study, the implant displayed a moderately rough surface with pits and grooves well identifiable at ESEM observation, the range was approx. 2–5 µm. Few CaP crystals from resorbable blasting procedures were detected using ESEM and semiquantitative EDX analysis (evidenced by limited traces of Ca and P), while they were not revealed by micro-Raman analyses [31].

SEM-FEG investigation allowed analysis of surface topography at higher magnifications. Interestingly, images at 200,000× original magnifications of the implant before immersion revealed a uniform nanorough surface, with well-identifiable irregular nanopits and nanogrooves, ranging from 30–100 nm. SEM-FEG also revealed the presence of limited crystal rods, identified as CaP crystals from EDX microanalysis. These data indicate that the surface showed a regular micro- and nanorough surface, in agreement with a previous investigation, which suggested a fractal architecture [13].

The surface nano topography of a biomaterial may strongly affect the cellular response [32,33,34,35,36].

Nanorough structures (1–100 nm) play an important role in the first moments after implant insertion, with regards to osteoblasts adhesion and protein adsorption [32,34,35].

Microrough structures (within 1–10 μm) were found to improve the interlocking between mineralized bone and implant surface and play a key role in mineralizing cells maturation and activity [36]. A previous study compared different titanium surfaces and showed a significantly higher osteoblast cell attachment and activity in presence of moderately rough surfaces when compared to a smooth untreated surface [37].

ESEM and SEM-FEG analyses revealed that the surface was markedly different after 28 days immersion in HBSS. A uniform mineral layer composed of microspherulites with 2–4 µm diameter that filled and covered the implant rough surface was detected. These data suggest a high reactivity of the surface upon exposure to biological fluids. EDX proved a fast, mainly superficial method able to semiquantitatively analyze precipitates in terms of atomic percentages (revealing a marked increase of Ca and P) [31].

Micro-Raman spectroscopy revealed that the microspherulites were mainly composed of B-type carbonated apatite. It may be observed that the spectra of the implant after immersion in HBSS were recorded at a higher laser power at the sample (i.e., 20 mW, Figure 4 spectrum b) than the implant before immersion (i.e., 10 mW, Figure 4 spectrum a); this choice was made to obtain stronger spectra, with higher signal-to-noise ratios, to obtain a more reliable characterization of the CaP phase. On the other hand, the spectra recorded on the implant at the same laser power at the sample (i.e., 20 mW, Figure 4, inset) showed sample degradation with formation of rutile, due to laser exposure. It is interesting to note that the latter phase was never detected in the spectra of the implant after immersion in HBSS obtained under the same conditions (Figure 4, spectrum b). This result suggests that the B-type carbonated apatite had a thickness sufficient to protect the titanium underneath from laser-induced degradation.

The micro-Raman results were further confirmed by XPS analysis. The XPS spectrum of the implant after immersion in HBSS is consistent with the formation of a B-type carbonated apatite and was free from Ti and TiO_2_ peaks. It must be recalled that XPS is a surface method (with ~10 nm penetration depth), so the latter behavior may be explained by considering that the nucleated CaP phase covered the implant surface completely, masking the signals from beneath.

Spectroscopic analyses showed that a highly biocompatible CaP (i.e., B-type carbonated apatite) formed on the implant surface. Actually, the mineral component of bone is primarily a carbonate substituted calcium apatite, where carbonate content is typically 2–8% by weight [38]. Carbonate may substitute into two anionic sites of the Ca_10_(PO_4_)_6_(OH)_2_ hydroxyapatite structure: at PO_4_^3−^ sites (B-type carbonated apatite) and OH^-^ sites (A-type carbonated apatite) [17,19,39]. Carbonate in bone mineral is primarily B-type; the fraction of A-type carbonate in biological apatites is very low. Actually, the carbonate in the A-sites has been reported to exert a greater predominant effect than B-type carbonate on the long-range order of apatite [40].

The result obtained in our study appears encouraging; actually, B-type carbonate apatite has been reported as an ideal artificial bone substitute because it is closer in chemical composition to bone mineral [41]. The biocompatibility of carbonated apatite may be related to the crystallinity decrease induced by the carbonate substitution, which increases solubility and, in turn, bone reformation or turnover [42].

The nucleation of a regular, homogeneous osteoconductive and biointeractive apatite layer provides a particularly suitable surface for osteoblasts, in terms of cell attachment and for new bone apposition, in particular for the presence of biologically active ions (such as Ca^++^) [17,24,25,26,39,43].

The transformation processes at the early phases from amorphous calcium phosphate to bone-like apatite has been recently observed at the nanoscale in an in-vitro model, describing amorphous calcium phosphate crystals that subsequently arrange in size very close to the needle-like crystals detected in our investigation (size 30–50 nm) [44]. These crystals will gradually transform to apatite crystals with elongated and platelet-like morphology [45]. The presence of a bioactive B-type carbonated apatite layer may well explain the histological finding and the gene expression recently reported for similar implant surfaces in animal and in laboratories studies [46,47,48,49,50].

## 5. Conclusions

The null-hypothesis was rejected, as in the present study we could find differences in superficial elemental composition in terms of higher amounts of Ca and P after HBSS immersion using EDX, XPS and micro-Raman spectroscopy.

This implant showed a micro-nano-textured surface supporting the formation of B-type carbonated apatite when immersed in HBSS. These properties may likely favor bone anchorage and healing by stimulation of mineralizing cells, revealing attractive characteristics to approach post-extractive implant placements and low-bone density area.

## Data Availability

Data sharing not applicable.
